# Digital Pathology in Hematopathology: From Vision to Deployment

**DOI:** 10.1111/ijlh.14533

**Published:** 2025-07-23

**Authors:** Ryan C. Shean, Anton V. Rets

**Affiliations:** ^1^ Department of Pathology University of Utah Salt Lake City Utah USA; ^2^ ARUP Laboratories Salt Lake City Utah USA

**Keywords:** digital pathology, image management system, whole slide imaging

## Abstract

Digital pathology (DP) has evolved alongside other technical advances, transforming our daily lives and diagnostic medicine. It is likely that, as in other areas of life, science, and medicine, the overall level of digitization will continue to rise, along with an increasing number of groups implementing DP. This review explores the clinical DP ecosystem with a focus on hematology and hematopathology, addressing the benefits of DP, such as improved workflow efficiency, remote practice, enhanced collaboration, and integration of artificial intelligence tools. Several challenges and pitfalls are also highlighted, such as technical scanner challenges, image management system issues, IT infrastructure, regulations, and the critical (and expensive) topic of data storage, and retrieval for DP. We also propose a roadmap for the successful implementation of DP, designed to support institutions of all sizes to make the transition to DP. This roadmap emphasizes well‐thought‐out, strategic planning and aims to ensure that organizations and individuals considering a switch to DP are able to deliver meaningful benefits to pathologists, health systems, providers, and most importantly, patients.

## 1 Introduction

The concept of digital pathology (DP) has been in development for over a quarter of a century. In 1986 Dr. Weinstein in his editorial defined “telepathology” as “the practice of pathology by visualizing an indirect image on a television screen rather than viewing a specimen directly through a microscope”. At that time, he acknowledged that telepathology was “at an embryonic stage of development” but it would “ultimately enhance the usefulness of the pathologist” [1]. During the last three decades, dramatic advances in technology and instrumentation have brought health care, including diagnostic medicine, into an era of digital innovation and transformation. Currently, DP can be defined as an “image‐based environment that enables the acquisition, management and interpretation of pathology information generated from a digitalized glass slide” [2]. The key event making DP a reality is the introduction of robust whole‐slide imaging (WSI) technologies that can be incorporated into clinical workflows. Additionally, the increasing sophistication of image analysis techniques and optimization of digital storage has made clinically useful DP implementations a possibility [3]. As a result, the field of pathology, including hematopathology and laboratory hematology, is undergoing a drastic paradigm shift in the way we handle and interpret microscopic images and other data. DP also creates new opportunities for data sharing and collaboration, with many other potential benefits for medical institutions, physicians, and patients.

Laboratory hematology has historically been ahead of anatomic pathology in some aspects of DP implementation. The field of hematology, in particular, has been heavily invested in the utilization of digital imaging since the first digital cameras became available [4]. The Food and Drug Administration (FDA) approved the first imaging system, DiffMaster Octavia by CellaVision, in 2001. Since then, many more digital systems have been developed and deployed into routine clinical practice. Hematology‐related ancillary test data (flow cytometry, molecular, etc.) have been relatively easy to digitize. However, large‐scale digital transformation involving entire anatomic pathology practices and, in some instances, entire healthcare systems, has revealed important roadblocks, including issues specific to hematopathology. Here, we will provide an overview of the clinical DP ecosystem and discuss some potential obstacles of DP implementation in hematology and hematopathology practice.

## 2 Benefits, Just a Few

Broadly speaking, the advantages of implementing DP fall into several categories:

1. Workflow optimization. Digitization of glass slides at the site of preparation and staining removes the need for transportation of physical slides. This can have a positive impact on turn‐around time (TAT) and monetary savings on courier services. Understandably, any TAT improvements would highly depend on the type of pathology practice. In an on‐site setting, TAT may increase with slide scanning. Alternatively, in our setting (a larger pathology/hematopathology department with an off‐site histology and immunohistochemistry laboratories) DP provides significant TAT reductions. Reference histology/immunohistochemistry laboratories can also transmit the WSIs to ordering pathologists much faster than physical slides. However, the most important factor determining any TAT benefit, even in small practices, is the successful implementation of the DP system as a whole [5].
2. Remote pathology practice [6]. The benefits of reviewing cases from off‐site locations became obvious during the COVID‐19 pandemic. But even after the pandemic, this technology broadens hiring options for pathology departments. Limited use of office space by pathologists who can sign out cases from their homes also has financial benefits by reducing overhead costs. In our institution, we have successfully implemented remote sign out of some services (flow cytometry, molecular, fluorescence in situ hybridization (FISH), etc.) by pathologists out of state. As hospital systems expand, being able to remotely access expert hematopathology services without needing space in new hospitals may also be seen as a benefit.
3. Ease of collaboration provided by remote communication and use of WSI. Concurrent WSI review and discussion can benefit pathology consultations (whether intra‐ or interdepartmental, or interinstitutional), teaching, and patient case discussions during treatment planning committees. For example, our hematopathology group now routinely scans cases sent for interinstitutional consultation, which allows us to reference priors much more easily. Furthermore, using WSIs in lieu of glass slides for such consultations could drastically reduce TAT for external clients.
4. Diagnostic augmentation via integration of artificial intelligence (AI). A rapidly increasing number of both internally developed and commercial deep learning algorithms are available, with even more in development. Although many focus on anatomic pathology, a few have been developed for hematology practice [7]; for example, DeepHeme is a recently developed bone marrow cell classifier that appears to approach human differential abilities in preliminary studies [8].
5. Research and innovation. Creation of datasets including WSI alone as well as integrating relevant clinical and laboratory information can provide fertile soil to expand our knowledge of diseases and therapeutic options. For example, our department is creating a database correlating available laboratory‐generated information including WSIs, flow cytometry, molecular test results, and final diagnosis as well as clinical outcome data to streamline the creation of AI applications.

## 3 Digital Pathology Systems: What to Consider

**FIGURE 1:**
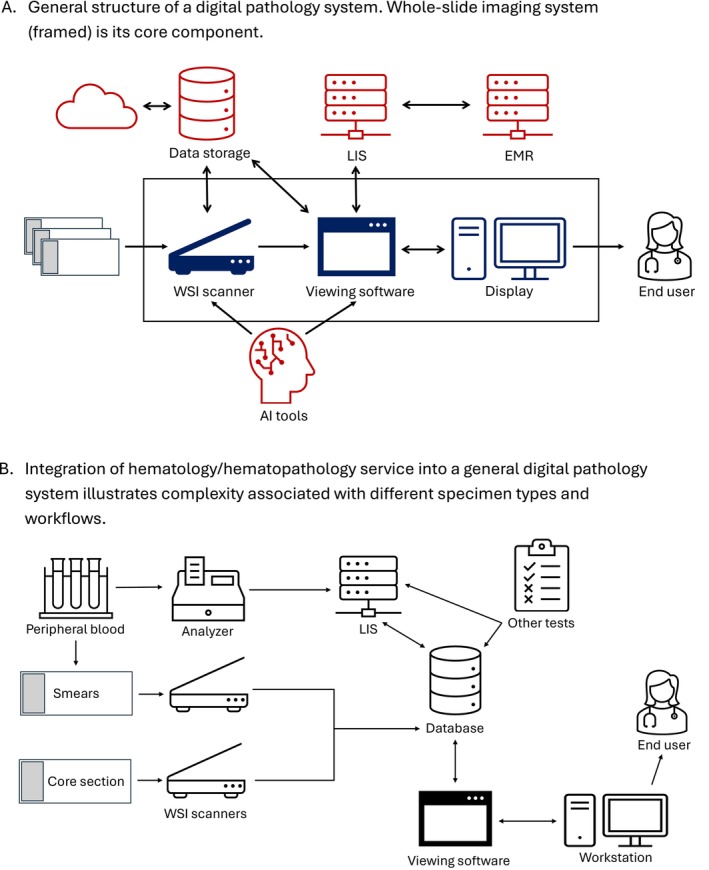
General structure of a digital pathology system. Whole‐slide imaging system (framed) is its core component. AI, artificial intelligence; EMR, electronic medical records; LIS, laboratory information system; WSI, whole slide image; Integration of hematology/hematopathology service into a general digital pathology system illustrates the complexity associated with different specimen types and workflows. Abbreviations: LIS, laboratory information system; WSI, whole slide image.

A DP system (DPS) is a structure that allows an end user (a pathologist, for example) to access and analyze patients' specimens and relevant clinical and laboratory data remotely. As such, the core capability of a DPS is conversion of a physical object—specifically, a patient's tissue on a glass slide—into a digital image that can be viewed, stored, and shared. Conceptually, this core, also referred to as “WSI system” consists of a WSI scanner, viewing software/image management system (IMS), and a calibrated monitor for review of WSI. Additionally, the WSI system can be integrated with the laboratory information system (LIS), electronic medical record (EMR), data storage/hosting, AI tools and algorithms, computer workstations, and network protocols for security and user authentication (Figure 1).

The complexity of the entire DPS and its integration requirements emphasizes the colossal effort required for successful implementation. This process demands a significant administrative, organizational, and financial commitment. Hematopathology departments, especially when they are a part of a larger pathology operation, should coordinate their efforts in digitalization with other relevant groups (e.g., surgical pathology) to pool resources and harmonize technologies.

### 3.1 WSI Scanners

The slide scanner is often the first thing that comes to mind when thinking of DP and is indeed a central component of any DPS. Its purpose is to transform physical tissue/smear slides into digital images. There is a wide variety of WSI scanners on the market. Continuous technological advancements lead to ongoing improvements of scanners' characteristics and specifications.

In general, tissue sections require scanning one level (or focus plane) producing a two‐dimensional image that can be represented in a Cartesian plane (perpendicular x and y axes). In hematology practice, this approach can suffice for sections of paraffin‐embedded tissues such as bone marrow cores, lymph nodes, etc. However, it is insufficient for peripheral blood smears and especially for bone marrow aspirate smears. Variations in thickness of liquid preparations and cellular overlap will cause many important features to be out of focus in a single plane. To visualize three‐dimensional structures and provide several focus planes, a scanner must be capable of “z‐stacking”. Z‐stacking, in turn, significantly increases scanning time and the size of the final image. Some scanners, developed specifically for hematology specimens (blood and aspirate smears) circumvent this limitation. “Traditional” cellular morphology platforms, such as CellaVision (Lund, Sweden), which is widely used for peripheral blood smears and some cytology specimens, capture individually focused snapshots of cells, instead of scanning the entire slide at one focus plane. Newer hematology imaging systems, such as Scopio (Scopio Labs, Tel Aviv‐Yafo, Israel), take a full‐field morphology approach utilizing computational photography and a physics‐based reconstruction model to produce high‐resolution images of peripheral blood and aspirate smears. Morphogo (Zhiwei, Hangzhou, China) takes images at several planes (z‐stacks) and then transforms them into a single two‐dimensional image. These three hematology‐focused platforms, however, operate as entire integrated digital systems as opposed to agnostic scanners that can be used for any purpose.

All‐purpose anatomic pathology scanners can encounter a variety of issues when used on hematopathology cases. Hematopathology service uses a variety of specimen types (i.e., peripheral smear, aspirate smear, and bone marrow core). Many scanners require scan settings to be pre‐set for each of these types. For example, a marrow core requires totally different scanner settings to capture high‐quality WSI than an aspirate smear. Options to ameliorate this include pre‐sorting and batching sample types, which can drastically increase personnel hours, or simply accepting some amount of scan failure and re‐scanning, which can increase TAT. Newer scanners may allow label scanning and per‐slide scanner settings. However, label specifications may be different from the current method of tracking and scanning hematopathology slides. When evaluating scanners, the most critical factor for the end user is the quality of WSIs they produce and their suitability for the intended purposes. Some factors to consider include the type of specimens and preparation techniques as well as the desired type of microscopy (e.g., bright field versus fluorescence). Objective measures of image quality to evaluate may include sharpness, contrast, color (as compared to the glass slides), focus, and effectiveness of z‐stacking. Subjective aspects important to hematopathology that may be assessed include ease of identifying minute morphologic features such as chromatin pattern, cytoplasmic granules, intracellular inclusions, microorganisms, mitotic figures, and ease of appreciating subtle lineage distinctions. Any scanner image evaluation must be performed (ideally on‐site) with slides produced in‐house and include all expected sample types for complex and routine cases (peripheral blood smear, aspirate smear, bone marrow core, lymph node with immunohistochemistry, etc.).

Many important technical and operational characteristics must also be considered. Even a “simple” parameter like throughput must be evaluated with several lenses—for example, real‐life speed of scanning, slide‐to‐slide transition time, total hands‐on time, how frequently hands‐on work is required, slide failure rate, out of focus scan rate, and others. Workflow parameters to consider include batch size, flexibility and scalability, degree of automation, batched versus continuous loading, error handling and communication, user‐friendliness of software and hardware, and availability of support and maintenance [9]. These factors, especially slide failure rates, must also be evaluated with each institution's specific slide preparation protocols, as manufacturer published specifications may not match true performance on an institution's actual slides. For example, in our on‐site demonstration of several slide scanners, the immersion oil residue on a coverslip (which is unusual for surgical pathology specimens) caused the scan failure rate to become unacceptably high. Other issues that caused high failure rates or totally out of focus WSI included labels or coverslips hanging off the edge of the slide, overlapping coverslips, or scratched coverslips.

Additional scanner attributes to consider include output features—for example, file format, any proprietary formats or standards, file size, frequency of file availability, and ease of network integration. Potential problems with scanner integration into the WSI/DPS and organizational network must also be carefully considered.

### 3.2 Image Management System (IMS)

As integral as image generation and viewing is to a DP workflow, the image management system (IMS) is also a critical component. Viewing software simply provides an interface to examine a digitized slide, and the core function of an IMS is to organize WSI for viewing. The most basic possible integration would simply be a folder containing all WSIs for the pathologist to find and review. However, in clinical hematopathology operations, it is often desirable for the IMS to have increased functionality. A more fully featured IMS might associate WSI with other metadata (patient information, case information, other associated slides (additional stains for example), or other meaningful information). IMS may offer organizational or workflow features, such as slide or case tracking, the ability to manage and authenticate users, assign cases to pathologists, or link cases to ancillary testing data. Further features that may be available include the ability to save slide annotations, report generating features, or integrated storage of other relevant case data, for example aspirate differential, dysplastic features, Hans' algorithm, c‐Myc expression, follicular lymphoma grade, number of ring sideroblasts, and others [10]. When selecting an IMS system, the above features must be considered alongside a variety of practical implementation factors. A factor to consider is ease of use for the pathologists—as features expand, so too does program complexity and potential for user frustration—a sometimes overlooked software factor that should be carefully considered.

Another important consideration is vendor‐specific versus vendor‐agnostic IMS. Most slide scanners will have a vendor‐specific IMS, but a variety of vendor‐agnostic systems are available. Vendor‐specific systems often use proprietary file formats and standards, which complicates interoperability and can negatively impact consult case compatibility [11]. Vendor‐agnostic solutions can typically handle multiple scanner brands and file types. This allows easier integration between other institutions/departments, including those with different scanners, and allows the department to select a wider variety of other DPS components and systems, including those from multiple manufacturers. However, even with vendor‐agnostic options, one should verify the degree of interoperability. The introduction of the DICOM format [12, 13], a vendor‐neutral file standard widely used in other areas of medicine, has been proposed to streamline compatibility across scanners, IMS, and WSI viewers. Some scanner‐neutral IMS vendors provide the proprietary WSI format software by default with an optional add‐on purchase of additional format readers. Considering whether the IMS of interest can process the original WSI file format without converting it to another format is important because format conversion is associated with significant computational burden, especially for z‐stacked images that are common in hematopathology, and may limit the use of some AI tools [9].

Many IMSs are primarily designed for either clinical or research use but not both. Clinical trials, which are extremely common at large academic hematopathology services, pose additional considerations. Trial protocols may require sending files to multiple centers with patient health information (PHI) stripped from them, the use of specific file formats, and compliance with more stringent data storage policies [14].

LIS integration is by far the most critical feature to evaluate. LIS integration usually comes in two flavors, either “unidirectional”, meaning the IMS only pulls data from the LIS but cannot modify the LIS, or “bidirectional”, in which both the LIS and IMS can modify each other. Although bidirectional LIS integration is more complex (and thus more expensive) to implement and maintain, it allows advanced features that would otherwise be extremely difficult to achieve. It is crucial that LIS and IMS vendors are involved in integration so that software and communication standards remain open and flexible [9].

Ease of IMS integration with third‐party tools is also important to consider. These tools may include report generating software, AI software such as automatic peripheral smear or aspirate differential tools, or integration with departmental workflows regarding complex cases such as ordering and interpretation of ancillary testing including flow cytometry, FISH, and others. Finally, WSI visualization, case navigation, and availability of other tools (e.g., annotation, side‐to‐side comparison between different stains etc.) can be very different depending on the vendor [15]. Thorough investigation of these technical capabilities of any proposed system is strongly advised.

### 3.3 Image and Data Storage

Given that many of the advantages of WSI revolve around easy access and transmission of slide files—data storage and retrieval become a key consideration. One hematopathology WSI, including Z‐stacking information, is about 1.5 GB—enough to watch an average 2‐h movie. Because of this large file size, storage, organization, and rapid retrieval of many WSI files become a non‐trivial undertaking very quickly. Before implementing a DP workflow, several key questions regarding data storage must be answered.

The first one, which significantly impacts system complexity and cost, is determining which WSI files will be retained. Since glass slides must be prepared for the scanning process, traditional archival storage of physical slides will meet current regulatory requirements. Therefore, one option is to only store WSI files during the diagnostic period. This approach is the simplest and cheapest; however, it prevents useful features such as rapid retrieval of previous cases, such as previous positive bone marrows or lymph nodes. Another option is storing only “key” diagnostic slides, which requires a more substantial archival and retrieval infrastructure. A final option is storing all WSIs, but as hematopathology cases almost always require multiple sample types and stains, sometimes in duplicate, this can cause total data storage requirements and the subsequent cost to expand extremely rapidly [16].

Another question is where and how WSIs are stored. Keeping files on‐site on local servers imposes a significant infrastructure build and future maintenance demands on IT staff. An alternative is cloud‐based or off‐site storage, but costs are often directly proportional to data retrieval speed. “Hot” or nearly instant retrieval is far more expensive than the cheaper “cold” or cheapest “glacier” storage, where data retrieval can take considerably longer. For example, assuming a hematopathology department digitizes three WSI per bone marrow case (peripheral smear, aspirate, and core biopsy) at 1.5 GB per WSI (no z‐stacking) and has a yearly volume of 2000 marrow cases, by the end of the year the total storage requirement for all these slides will be 9 TB. Using a popular commercial vendor's publicly available pricing information (https://aws.amazon.com/s3/pricing), the cost to store this much on an instant retrieval database would be $207 per month. This same storage on glacier storage (designed to be accessed once or twice per year) would be $8.91 per month. However, on top of simple storage costs, cloud storage providers may charge upload and retrieval fees which often are much higher for glacier storage than instant storage. There may be additional fees for indexing requests and searches of the database. Therefore, a clear understanding of required access volumes and frequency is paramount, and contract negotiation with any cloud storage provider is extremely important.

A final consideration for WSI storage is backup strategies. Extremely long‐term backup storage of WSI files on cheap, alternative media such as optical disks or tape is one option. Some organizations do not digitally back up at all due to the retention of glass slides. However, if re‐digitizing a large body of slides becomes necessary, this could rapidly overtax scanning personnel and infrastructure.

Perhaps counterintuitively, in almost any DP implementation, the cost of the machinery itself (which is a fixed up‐front cost) will often become trivial compared to the monthly cost of WSI storage, retrieval, and backup. Especially since without a clearly defined deletion policy, per‐month storage costs will inexorably increase month over month as total database size grows. In the example above, the cost at month 13 would be $207 and at month 120 would be 10 times that. Memorial Sloan Kettering Cancer Center has published a detailed financial analysis of their DP operations including exact cost, and had an annualized data storage cost of ~1.6 million dollars per year [17]. Careful consideration of storage specifications and pricing factors are incredibly important to the long‐term future of the DP program.

### 3.4 AI Applications

While surgical pathology AI applications have received widespread attention [18], AI tools for cellular analysis in hematology have a comparatively long history [19]. AI‐based automatic peripheral blood differential applications have been around since the early 2000s, and the CellaVision platform has been incorporated onto leading analyzers since and has become the de‐facto industry standard. More recently, competitors such as Mindray have emerged as an alternative, with recent studies showing comparable performance to leading brands [20] and several novel features, such as automatic detection of malaria‐infected erythrocytes [21, 22]. However, tissue‐based AI applications in hematopathology have a relatively low representation compared to surgical pathology.

Due to the variety of different systems required for a DP workflow, AI applications can be deployed on each individual part of the workflow. Scanner manufacturers, for example, are developing “app store” models, which could enable “edge scanning”, allowing the AI model to begin working during the scanning process. Alternatively, post‐scan integration of AI tools can be deployed onto the IMS, the viewing software, or as a standalone program. One software package that deserves special attention is QuPath, which is an open‐source platform to develop, share, and implement new algorithms and models for tissue analysis [23]. As AI can integrate at a wide array of touchpoints, determining where and how potential AI systems may fit into a planned DP implementation is extremely important.

A particular area of interest in hematopathology is using AI for more repeatable and quantitative assessment of commonly used semi‐subjective parameters. One such application is the evaluation of bone marrow biopsy cellularity, with the MarrowQuant 2.0 model being developed and publicly available on QuPath [24], and other groups developing algorithms to more reproducibly assess cellularity in myeloproliferative neoplasms [25]. Another subjective parameter with AI applications developed is bone marrow fibrosis [26]. In some cases, the use of AI may allow increased granularity in reporting and prognosis of these parameters, such as refining fibrosis scoring beyond the traditional 4‐tier system. Additionally, AI algorithms could uncover other, previously unknown, stromal changes that may have diagnostic or prognostic significance [27].

Another promising AI application is automating the time‐consuming task of an aspirate differential count, with commercially available digital imaging systems [28, 29], and a standalone deep learning algorithm already described in the literature [8]. As briefly mentioned earlier, both the Scopio labs X100 and the Morphogo systems are commercially available products with automatic digital analysis of aspirate smears available [30]. While both products are capable of providing an automatic differential [31] and both products are CE marked for use in the EU, at the time of writing, only the Scopio instrument has received FDA approval for diagnostics in the United States [32]. However, just like any other laboratory test or instrument—careful verification and validation on‐site are absolutely necessary.

Finally, AI in hematopathology can be used for much more than simple image analysis and classification. Recent studies have shown that deep learning‐based analysis can predict clinically relevant genetic aberrations directly from stained bone marrow aspirates [33]. Future work in this area is likely, with the potential to cheaply and quickly screen patients for therapy‐relevant alterations directly from a digital smear. One of the most exciting applications of AI is the integration of multi‐modal data streams, including digital images, with other factors such as patient information or ancillary testing results. Various models that integrate multiple data types have been developed, including the prediction of post‐transplant outcomes, the need for treatment in patients with chronic lymphocytic leukemia [34], and the grading lymphoma [35].

The potential of AI uses in hematopathology is continuously expanding, although ensuring proper validation and regulatory compliance will remain extremely important. Depending on intended function, some AI tools augment the diagnostic workup, which may place them under regulatory oversight. Other AI applications may focus on control or workflow improvements, which may not fall under a strict regulation.

## 4 Regulations

Regulations regarding DP and AI are in a constantly evolving flux. In the United States, oversight is primarily through the FDA, which regulates DPS as medical devices [36]. WSI systems (FDA product code “PSY”), by the FDA definition, consists of “(a) an image acquisition subsystem that converts the content of a glass slide into a digital image file, and (b) a workstation environment for viewing the digital images”. “Image acquisition” is equivalent to a WSI scanner. Workstation environments include an “image review manipulation software” (FDA product code QKQ), a “computer environment”, and “display” (FDA product code “FZZ”) [36].

WSI systems are typically regulated as Class II devices, and new devices can be submitted for approval through the 510(k) regulatory pathway. Additionally, approval has been granted for other components of a DPS including image viewers, IMS, display devices, and classification decision support systems [37]. Currently, among six WSI devices that have FDA clearance, two are hematology‐focused: CellaVision DC‐1 and Scopio X100 [38]. For the most up‐to‐date information on regulatory approvals in the US, the FDA maintains a full list of approved AI enabled medical devices [39].

In the EU, DP systems are regulated under EU 2017/746 (IVDR) [40, 41]. Just like in the US, devices are classified by risk, and evidence of clinical safety and performance must be demonstrated.

Regardless of regulatory status, laboratories must internally validate their DPS. Best practice guidelines have been issued by several agencies and associations including the College of American Pathologists [42], Digital Pathology Association [43], the European Union [44], the Canadian Association of Pathologists [45], the Royal College of Pathologists [46], and others. The College of American Pathologists, in collaboration with the American Society for Clinical Pathology and Association for Pathology Informatics, recommends that validation/verification include cases that reflect the spectrum of complexity of specimen types and processing, staining, and diagnoses as appropriate. The purpose of validation is to ensure high (> 95%) diagnostic concordance between the WSI system and glass slides [42].

## 5 Fiscal Performance

As there are extremely significant capital expenditures and almost certainly increased operating expenses, the main roadblock to DP implementation is its cost. There are only a few publications discussing the business model of DP, mostly in large academic institutions or cancer centers, and specific data on hematopathology services is even more scarce. However, the Digital Pathology Association has published a comprehensive review including a cost estimator tool [47]. Careful analysis of projected operational costs and return on investment associated with the transition to DP from the perspective of the laboratory/pathology department and healthcare system must be performed very early on in the planning process.

The principal cost categories are capital expenditures and ongoing costs. Capital expenditures include the purchase of hardware (scanners, monitors), software including IMS, and IT infrastructure. Ongoing costs include operating and maintenance costs: labor (staff), space, storage, maintenance contracts, and others. Whereas capital costs are generally exclusively fixed, some ongoing expenses are fixed (maintenance contracts and space) or variable (data storage, servers). Generally, increasing the volume of scanning (scanning more slides, eliminating scanner downtime) results in decreased fixed costs per scanned slide despite an expected increase in the variable costs. To decrease overall costs, institutions may consider some options. For example, leasing a scanner (and paying per scanned slide) may be a better financial option than purchasing the scanner in some situations. Bundling service contracts may also provide a lower cost. Automated scanner feeding may require fewer supporting staff and reduce labor costs.

It is predicted that the cost‐effectiveness of DP is more likely achieved by complete transition from glass slides to DP. Alternatively, partial implementation of DP, when both WSI and glass slides are available for review, creates duplication of workflows and increases overall costs compared to a “glass only” or “digital only” approach. This phenomenon must be anticipated in institutions planning a gradual step‐by‐step implementation, for example, when DP switching involves one pathology service at a time.

Of particular importance is the cost of data storage. Operational cost analysis of Memorial Sloan Kettering Cancer Center (New York, USA), a large academic cancer center, showed that digital storage expenditures increased exponentially over a 5‐year period. The growth of digital storage expenses overwhelmed other operational costs, such as vendor service, personnel, hardware, software, and IT [16]. These costs may be potentially alleviated by choosing the slide retention approach discussed above that would be most reasonable and cost‐effective for the institution.

The current DP‐associated reimbursement strategies in the United States do not provide direct payments. Digitalization‐related codes, approved by the American Medical Association (AMA) belong to Category III. This category is not associated with a payment rate or direct reimbursement by the Centers for Medicare and Medicaid Services (CMS) but is designed to document the utilization of the coded service and support the potential creation of Category I codes in the future. There are currently 43 Category III codes associated with DP (13 created in 2023 and 30 in 2024) [48]. As there is no direct billing for DP, any return on investment must derive primarily from the elimination of waste and gains in productivity.

It must be emphasized that a simple replacement of traditional microscopy by digital review of WSI may not create a direct benefit. It has been shown that pathologists may spend more time reviewing WSIs compared to glass slides. Hanna et al., for example, showed a 19% decrease in efficiency using WSI with no difference by subspecialty, reader, or specimen type [49]. It is reasonable to assume that this issue may be exacerbated by the complex and detailed cytomorphology required in hematopathology. However, use of assisting tools, such as AI‐based cell classifiers for blood or aspirate smears, may increase efficiency.

At our institution, projected monetary benefits of DP derive mainly from cost savings associated with workflow optimization and increased operational efficiencies. The remote location of histology and immunohistochemistry laboratories from many pathologists' offices results in prolonged transportation time for glass slides; this issue is exacerbated by multiple rounds of stains across multiple days required for complex cases in hematopathology. This inefficiency is easily eliminated by DP. By the same token, decreased demand for slide filing and retrieval, as well as transportation to and from the remote slide storage facility, cuts the amount of manual labor and time. Ease of WSI retrieval for retrospective review, education, and interdepartmental conferences improves pathologists' job satisfaction [17]. Another major benefit is the ability to provide diagnostic services remotely, which may positively affect academic productivity, recruitment efforts, and improve occupational well‐being. Remote diagnosis is particularly relevant for highly specialized hematology services and could allow expansion of expert services to underserved communities worldwide. A caveat of diagnostic off‐site services is that it requires IT infrastructure optimization including bandwidth, compatibility of software between the central and remote sites, safety and security protocols, appropriate hardware (monitors, etc.), and compliance with regulations [6].

## 6 Implementation Roadmap

We are at a dawn of the digital era; evidenced by the progressive and accelerating percolation of digital technologies into all aspects of our lives. Our laboratories, hospitals, and whole healthcare systems are not exempt from this irreversible change. Most likely, the digital transformation of diagnostic medicine, including laboratory and pathology practices, is just a matter of time. The task of introducing and implementing DP in clinical practice is colossal. Not only does it change our laboratory operations, but it also creates a major shift in how the end users access, review, and integrate information to make diagnoses.

So, where to start?

1. DP transformation starts with a vision, which is rooted in the current state of the laboratory and is aligned with the short‐ and long‐term goals of the laboratory/department/healthcare system. It is important to evaluate your laboratory's current practices and identify clear goals that DP can help you achieve. Alignment of these goals with those of your institution or healthcare system will further favor their successful accomplishment.
2. After a clear vision is formulated, a workable strategy must be developed. This includes identifying your intended DP applications (clinical workflow, research, education, etc.) as well as whether DP deployment will involve the entire laboratory/department at once or will be a longer step‐by‐step implementation. An advantage of an all‐at‐once approach is a higher likelihood of achieving cost‐effectiveness. However, there is a larger initial investment and the risk of significant unexpected roadblocks including dissatisfaction of the end users. According to the literature, a gradual digital transition is the most common approach, especially in larger institutions, including our center. This transition can be subspecialty‐dependent (e.g., surgical pathology subspecialties first, then hematopathology), test‐dependent (e.g., flow cytometry followed by FISH, smear morphology, then tissue slides), or service‐dependent (outside consultations followed by internal cases). Gradual implementation has smaller incremental risks but creates at least temporary workflow redundancies resulting in increased costs. Depending on the type of specimens and preparation, one should decide if your digital workflow will include smears (peripheral blood, bone marrow aspirate, or even cytology preparations). Additionally, other, less tangible factors must be considered, such as your department's culture, mindset, openness to change, and other potential organizational problems. A tool that may be helpful for analyzing these factors is “strength‐weaknesses‐opportunities‐threats (SWOT) analysis” [50]. Careful evaluation of the current laboratory processes by creating maps and workflow charts will provide a direct visual concept which can be used to introduce anticipated DP‐related changes as alternative process maps. Contingency plans should also be developed early.
3. Conversion of ideas into action requires involvement and buy‐in from administration. Having a clear proposal, SWOT analysis, being consistent, persistent, and creating a sense of urgency are some of the key components to help convince senior leadership to provide support for the project.
4. Digital transformation is a team effort. The effectiveness of your team is key to success. Thoughtful and careful consideration should be given to the members of the team. The team must include (a) decision‐makers (executives) including members of administration, (b) leaders in relevant non‐pathology services as advisory members, and (c) end users (pathologists, trainees, laboratory personnel, customers). Key services to include are operations, quality, IT, compliance, legal, purchasing, financial, etc. The overall complexity of a DP migration will also require a designated project manager. Considering how lengthy the process can be, a project manager should be skilled at engaging team members, keeping focus on priorities, and helping team members understand and be accountable for their roles.
5. Evaluation and selection of hardware and software is a lengthy and energy consuming process. The importance of close interaction with vendors including extensive hands‐on on‐site demonstrations and engaging internal subject matter experts must be emphasized. The inclusion of all appropriate personnel, such as laboratory scientists, hematopathologist, IT specialists, scanning staff, quality and compliance team members is essential for addressing critical points and identifying potential problems ahead of time. Close attention should be paid to the interoperability of the offered products and the de facto status of integration of the considered IMS with your LIS.
6. The “purchase, install, validate” phase must be aligned with education and training of your personnel and end users. Introduction of DP in clinical service may (not uncommonly) meet strong resistance from end users, which is a significant limiting factor that affects the dynamics of the whole unit. There are a variety of strategies to help mitigate this. Focusing on healthcare professionals, Nilsen et al. suggested that the influence to change, feeling prepared for the change, and recognition of the benefits of change including a perception of the benefits for patients are key factors to increase the likelihood of a successful transformation [51]. As a leader, one must communicate a clear message to the group, be attentive and open to their concerns, and exercise effective and purposeful communication before, after, and during a DP implementation.
7. As the new workflows are deployed, regular assessments to identify and measure benefits as well as detect and resolve problems must be undertaken.

## 7 Future at a Glance

As DP practice gains more widespread adoption, it is exciting to prognosticate on the potential future of a wholly DP ecosystem. For example, the Swiss Digital Pathology Consortium is creating a national and centralized DP infrastructure [52]. As well as the benefits that may be expected for communication and diagnosis of patient samples, the vision is to integrate this data to allow large‐scale pooling of health data for research purposes and clinical trials. Our department is creating a multi‐modal hematopathology database for use in clinical and basic research that organizes and integrates digital morphology with all other relevant diagnostic information. Creation of such high‐quality infrastructures and data sets will allow researchers to more easily realize advancements and democratize precision medicine for the betterment of patients worldwide.

## 8 Conclusion

The digital transformation of our society necessitates a paradigm shift for diagnostic medicine, including hematology and hematopathology services. As generation of WSI has become more available for anatomic pathology, DP deployment at pathology practices of all sizes has grown drastically in the last 10 years. Although the hematology community has more experience with digitizing data and workflows than anatomic pathology, a broader spectrum of slide preparations has created obstacles for full digital transformation of hematology and hematopathology. As technology advances, more ways to overcome these obstacles are available and marketed to departments. In the current environment, full digitization of hematology services and deployment of AI technologies is a real possibility. However, as a hematology community, we must learn how to properly evaluate and discuss this upcoming digital change to better serve our patients.

## Ethics Statement

The authors have nothing to report.

## Consent

The authors have nothing to report.

## Data Availability

Data sharing is not applicable to this article as no new data were created or analyzed in this study.
